# The Neural Stem Cell Properties of PKD2L1^+^ Cerebrospinal Fluid-Contacting Neurons *in vitro*

**DOI:** 10.3389/fncel.2021.630882

**Published:** 2021-03-15

**Authors:** Shuo Wang, Yuqi He, Huiqian Zhang, Li Chen, Liang Cao, Leiluo Yang, Chunqing Wang, Yujie Pan, Qian Tang, Wei Tan, Xiaowei Dou, Qing Li

**Affiliations:** ^1^Department of Orthopedics Traumatic, The Affiliated Hospital of Guizhou Medical University, Guiyang City, China; ^2^School of Clinical Medicine, Guizhou Medical University, Guiyang, China; ^3^Shandong Juxian People’s Hospital, Rizhao City, China; ^4^Clinical Research Center, Affiliated Hospital of Guizhou Medical University, Guiyang City, China

**Keywords:** cerebrospinal fluid-contacting neurons, neural stem cell, PKD2L1, cervical spinal cord, mice

## Abstract

Cerebrospinal fluid-touching neurons (CSF-cNs) exist in the region surrounding the central canal of the spinal cord, which locate in the adult neurogenic niche. Previous research showed that CSF-cNs expressed the molecular markers of immature neural cells *in vivo*. Here, we explored the potential of CSF-cNs as neural stem cell *in intro*. We first found that PKD2L1^+^ CSF-cNs, isolating by FACS using the molecular marker PKD2L1 of CSF-cNs, expressed neural stem cells markers like Nestin, Sox2, and GFAP by immunofluorescence staining. PKD2L1^+^ CSF-cNs were able to form neurospheres and passaged *in vitro*. Immunofluorescence staining showed that the neurospheres forming by PKD2L1^+^ CSF-cNs also expressed neural stem cell markers Nestin, Sox2 and GFAP. The neurospheres expressed proliferation markers Ki67 and PCNA by immunofluorescence staining, indicating that the neurospheres forming by PKD2L1^+^ CSF-cNs were proliferative. The neurospheres, forming by CSF-cNs, had the ability of differentiation into neurons, astrocytes, and oligodendrocytes. Collectively, our data suggested that PKD2L1^+^ CSF-cNs have the properties of neural stem cells *in vitro* and may provide a promising approach for the repair of spinal cord injury.

## Introduction

Historically, the mammalian central nervous system has been considered to remain generally unchanged during adulthood. However, recent research has confirmed that the mammalian central nervous system contains endogenous neural stem cells (NSCs) that exhibit self-renewal properties and are able to undergo multi-directional differentiation (Reynolds and Weiss, 1992). Cells derived from NSCs can produce new neurons, astrocytes, and oligodendrocytes, and therefore maintain the dynamic balance of the nervous system. The discovery of NSCs overturned our previous belief that the central nervous was unable to regenerate and provides significant new hope for the treatment of neurodegenerative diseases, spinal cord injuries, and other neurological diseases. Although research has shown that endogenous NSCs are located in specific niches within the dentate gyrus of the hippocampus and the central ependymal area of the spinal cord (Doetsch et al., 1999; Seaberg and van der Kooy, 2002; Hugnot and Franzen, 2011; Obernier et al., 2018), the exact composition of these NSCs have yet to be determined. This is because of the complex composition of cells in these areas, and due to the fact that cellular markers often overlap with each other.

Previous research referred to ependymal cells located in the NSC niche as neural stem cells (Redmond et al., 2019). However, recent research involving single cell transcriptomics and fate mapping found that ependymal cells did not exhibit the same functionality as NSCs (Shah et al., 2018), thus creating further confusion in the field. Around the same time, we noticed another special cell type, referred to as cerebrospinal fluid-contacting neurons (CSF-cNs) (Vigh et al., 1977; Kutna et al., 2014; Orts-Del’Immagine et al., 2017). These cells are predominantly distributed in the ependymal layer of the central canal of the spinal cord, the parenchyma surrounding the spinal cord, and the periaqueductal gray matter at the junction of the midbrain and the pons (Vigh et al., 1977; Djenoune et al., 2017). One side of these cells protrudes to connect with the cerebrospinal fluid while the other side links with the parenchyma of the spinal cord, thus forming the “brain-cerebrospinal fluid barrier” (Zhang et al., 2003; Jalalvand et al., 2016).

Huang et al. (2006) found that polycystic kidney disease type 2 channel 1 (PKD2L1) is a specific biomarker of CSF-cNs. Subsequent studies also supported the fact that PKD2L1 channel proteins can specifically label CSF-cNs (Djenoune et al., 2014; Kutna et al., 2014; Orts-Del’Immagine et al., 2014; Sternberg et al., 2018). The discovery of specific markers for CSF-cNs has facilitated our ability to investigate these special cells. More recent studies have identified that CSF-cNs express certain immature neuronal markers, such as adrenocortical hormone (DCX), polysialic acid nerve cell adhesion molecule (PSA-NCAM), Sox2, and β-IIItubulin/Tuj1; as well as some mature neuronal markers, including NeuN and MAP2 (Kutna et al., 2014; Orts-Del’Immagine et al., 2017). CSF-cNs also exhibit the electrophysiological characteristics of immature neurons (Orts-Del’Immagine et al., 2017). These markers and characteristics persist in CSF-cNs into adulthood (Stoeckel et al., 2003; Marichal et al., 2009; Sabourin et al., 2009; Petracca et al., 2016). Collectively, these findings indicate that CSF-cNs maintain a persistently low state of differentiation during development and maturation in mammals. We have previously shown that CSF-cNs affect the expression of endogenous neural progenitor cells and the recovery of neural function after spinal cord injury (He et al., 2020). Therefore, we hypothesized that CSF-cNs exhibit the characteristic properties of NSCs.

In the present study, we focused on neonatal mice and successfully isolated and purified primary CSF-cNs from the tissue surrounding cervical spinal cord central canal by flow cytometry. CSF-cNs were purified and then cultured adhered to the wall and in suspension, respectively, we then determined the expression of a range of NSC markers. We found that CSF-cNs expressed known markers for NSCs and could proliferate and differentiate into neurons, astrocytes, and oligodendrocytes. These findings demonstrated that when cultured *in vitro*, CSF-cNs exhibit the potential for self-renewal and multi-directional differentiation. Our findings provide a new direction for investigating the potential for liquid contact neurons to exhibit NSC potential and thus promote nerve cell regeneration and repair of the nervous system following spinal cord injury.

## Materials and Methods

### Animal Model

All neonatal female C57BL/6 mice were provided (within 24 h of birth) by the Experimental Animals Center of Guizhou Medical University [Experimental Animal License Number: SCXK (Qian) 2012-0001]. All animal experiments were conducted in strict accordance with the National Institutes of Health Guide for the Care and Use of Laboratory Animals and were approved by the Animal Care Ethics Commission of Guizhou Medical University.

### Isolation of Primary Nerve Cells From the Cervical Spinal Cord of Mice

**FIGURE 1:**
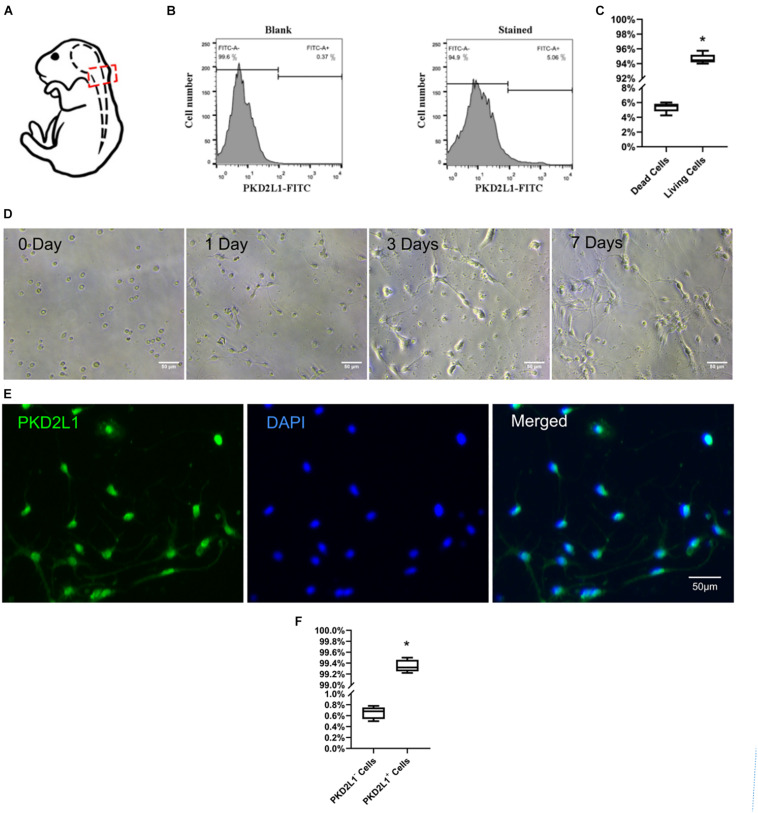
Isolation, culture, and identification of cerebrospinal fluid-contacting neurons (CSF-cNs). **(A)** A model showing how CSF-cNs were extracted. **(B)** Fluorescence-activated cell sorting (FACS) analysis of CSF-cNs from the micro-dissected CC zone. **(C)** The ratio of living/dead cells after FACS. *P < 0.05 verse the dead cells group. **(D)** FACS-isolated CSF-cNs were cultured from day 0 to day 7. **(E)** Immunofluorescence analysis (after FACS) on day 7 showing that CSF-cNs expressed polycystic kidney disease type 2 channel 1 (PKD2L1). **(F)** The ratio of PKD2L1^+^/PKD2L1^–^ cells on day 7 after FACS. *P < 0.05 compared to PKD2L1^–^ cells group.

Primary cultures of nerve cells were prepared from the neonatal female C57BL/6 mice (within 24 h of birth) in accordance with established methods (Brewer and Torricelli, 2007; Walker and Kempermann, 2014; Mothe and Tator, 2015; Belenguer et al., 2016), with some modifications. First, the neonatal 10 mices were cleaned with 70% ethanol. We then opened their skulls to collect the brains (Figure 1A). For dissection, the brains were placed in a Petri dish containing pre-cooled eagle’s medium nutrient mixture-high glucose [DMEM/HG (Gibco, Carlsbad, CA, United States)] under a stereomicroscope and the tissue surrounding cervical spinal cord central canal was isolated using ultra-fine forceps. The target tissue block was repeatedly and evenly cut into 1 mm^3^ pieces with sterile ophthalmic scissors in an ice box which was kept motionless for 2 min. The precipitated tissue fragments were then aspirated into a new pre-cooled 15 ml centrifuge tube with a sterile straw. We then added 5 ml of pre-heated papain solution (Sigma, Louis, MO, United States) for 10 min; the solution was then placed into an incubator at 37°C for 30 min. The digestive solution was shaken gently once every 5 min. Following digestion, we added an equal amount of inoculum which includes serum-free eagle’s medium nutrient mixture-F12 [DMEM/F12 (Gibco, Carlsbad, CA, United States)] supplemented with 2% B27 (Gibco, Carlsbad, CA, United States) to stop the digestion. The tissue block and the 15 ml centrifuge tube were then centrifuged at 1,500 rpm for 5 min. The supernatant was discarded and inoculation fluid was re-added and re-centrifuged. Following centrifugation, the supernatant was discarded and 1.5 ml of fresh inoculum was re-added. The tissue blocks were gently immersed in the instillation fluid using a 2 ml sterile glass straw; the blocks were immersed in the fluid slowly by blowing with the glass straw 10 times (for 40 s each time). During this procedure, it was important not to create any bubbles. After blowing, the tissue blocks were placed on ice and allowed to rest for 2 min. Then, 1.5 ml of supernatant was drained into a 15 ml centrifuge tube and placed on ice. Next, we added 1.5 ml of fresh inoculum to the precipitated tissue mass and used the straw to blow inoculum over the tissue (this procedure was repeated three times). Finally, the residual tissue was discarded, leaving approximately 6 ml of cell suspension. The cell suspension was filtered with a 200-mesh cell screen to remove the undigested tissue fragments and filtered single-cell suspension was collected in a 15 ml centrifuge tube. Subsequently, cells were suspended in culture medium which includes serum-free DMEM/F12 (Gibco) supplemented with 2% B27 (Gibco), 20 ng/mL bFGF (Proteintech, Rosemont, IL, United States), and 20 ng/mL EGF (Gibco, Carlsbad, CA, United States).

### Sorting of PKD2L1^+^ Cells by Fluorescence-Activated Cell Sorting

The cells were prepared from 10 neonatal female C57BL/6 mice as above description and filtered by a 40 μm strainer (352340, BD, United States). Cells were fluorescently labeled with the specific CSF-cNs marker PKD2L1 and then screened by fluorescence-activated cell sorting (FACS) (Figure 1B). Dispersed cells were counted in medium at a concentration of 1 × 10^6^ cells/mL; all counting procedures were undertaken on ice. Rabbit anti-PKD2L1 primary antibody (Millipore Sigma, Burlington, MA, United States) was added to the cell suspension and incubated for 45 min. The cell suspension was then centrifuged for 5 min at 1,000 rpm. The primary antibody solution was then discarded, and the cells were washed three times with PBS. Cells were then resuspended using FACS incubation solution. FITC-conjugated goat anti-rabbit secondary antibody was then added and incubated for 30 min. The cell suspension was centrifuged for 5 min at 1,000 rpm. The secondary antibody solution was then discarded, and the cells were washed three times with dissection solution. Finally, cells were resuspended using dissection solution, placed in a FACS Aria III flow cytometer (BD Biosciences, San José, CA, United States), and PKD2L1^+^ cells carrying green fluorescence (FITC) were identified. We used a wavelength of 488 nm to detect the fluorochromes used in this protocol. The sample station and collection module were cooled to 4°C during FACS. The PKD2L1^+^ cell population was analyzed by FlowJo software. The control group featured a single-cell suspension that was devoid of any antibody.

### Detection of Cell Viability

The viability of the PKD2L1^+^ cells was determined by trypan blue staining (Gibco, California, United States). Following the completion of FACS, 10 μl of single-cell suspension was removed and added to 10 μl of 0.4% trypan blue solution; the solution was then mixed thoroughly. Next, 10 μl droplets of suspension were removed and placed onto the cell blood count (CBC) board. We then used microscopy to determine the cell survival rate.

### Petri Dish Pretreatment

Petri dishes were coated with poly-L-lysine prior to adherent culture. The poly-L-lysine (100 g/ml) was added dropwise on to the Petri dish. Incubation was carried out overnight at room temperature, washed with PBS twice, and 95% of the liquid was then removed. The culture plate was then placed on a clean table and air-dried for more than 1 h for subsequent use. This step was omitted whenever the neurosphere-forming assay was performed.

### Neurosphere-Forming Assays

On day 3 of culture, CSF-cNs were digested by papain and transferred to a Petri dish for further culture but without a poly-D-lysine coating. All cells were cultured in a special neurosphere medium (Neurobasal-A supplemented with 2% B27, 0.5 mM L-Glutamine, 20 ng/mL bFGF, and 20 ng/mL EGF) in 5% CO_2_ and 20% O_2_ at 37°C. Half of the medium was refreshed every 2 days (Minamino et al., 2015).

### Immunocytochemistry

Cells were washed once with 1 × PBS and fixed with 4% paraformaldehyde at 37°C for 15 min. The cells were then permeabilized with 0.5% Triton X-100 in 1 × PBS for 10 min, and then incubated with 5% normal goat serum (Absin, Shanghai, China) for 1 h at room temperature to block non-specific binding sites. The cells were then incubated with the following primary antibodies (diluted in 1% normal goat serum) in a humidified chamber at 4°C overnight: rabbit anti-PKD2L1 1:500 (Millipore Sigma), mouse anti-Nestin 1:200 (Proteintech, Rosemont, IL, United States), anti-Sox2 1:200 (Proteintech, Rosemont, IL, United States), anti-GFAP 1:200 (Proteintech, Rosemont, IL, United States), anti-Ki67 1:200 (Proteintech, Rosemont, IL, United States), anti-PCNA 1:200 (Proteintech, Rosemont, IL, United States), anti-Tuj1 1:200 (Proteintech, Rosemont, IL, United States), anti-NeuN 1:200 (Millipore Sigma, Burlington, MA, United States), and anti-O4 1:200 (Proteintech, Rosemont, IL, United States). The next morning, the cells were washed three times with 1 × PBS for 5 min at 37°C. Once the unbound primary antibody had been completely removed, the cells were incubated with an appropriate secondary antibody for 1 h at room temperature in the dark; we used two secondary antibodies: Alexa Fluor 594 goat anti-mouse IgG or Alexa Fluor 488 goat anti-rabbit IgG 1:500 (Thermo Fisher Scientific, Waltham, MA, United States). The cells were then washed three times for 5 min with 1 × PBS. After immunostaining, the cells were incubated with DAPI (Thermo Fisher Scientific, Shanghai, China) for 2 min in the dark to label the nuclei. For the negative control, the primary antibody was replaced with PBS buffer. A fluorescence microscope (Observer AI, Carl Zeiss AG, Germany) was used to visualize all immunostaining. The counting function in Photoshop CS3 (Adobe, San Jose, CA, United States) was then used to count the numbers of PKD2L1^+^/Nestin^+^, PKD2L1^+^/Nestin^–^, PKD2L1^+^/SOX2^+^, PKD2L1^+^/SOX2^–^, PKD2L1^+^/GFAP^+^, and PKD2L1^+^/GFAP^–^ double-positive cells, and Image J (NIH, Bethesda, MD, United States) was used to quantify the mean fluorescence intensity. Mean fluorescence intensity = integrated density/area; percentage immunopositive cells = 100 × number of immunopositive cells/total number of cells (DAPI-stained cells) (Li et al., 2015).

### Statistical Analysis

All experiments were done in triplicate. ImageJ was used to count cell numbers. Data were expressed as the mean ± SEM and analyzed using Student’s *t*-test by Prism 8. *P* < 0.05 was considered statistically significant.

## Results

### High Purity CSF-cNs Were Successfully Cultured *in vitro*

Nerve cells were acquired from the tissue surrounding cervical spinal cord central canal and sorted by fluorescent-activated cell sorting (FACS) (Figure 1B). The proportion of cells surviving after FACS was 94.63 ± 0.59% (Figure 1C). The cells were then seeded onto poly-D-lysine-coated culture plates and cultured for 7 days prior to use (Figure 1D). The purity of the CSF-cNs was approximately 99.34 ± 0.10%, as determined by immunofluorescence staining with the specific marker PKD2L1 (Figures 1E,F).

### CSF-cNs Expressed Markers for Neural Stem Cells

**FIGURE 2:**
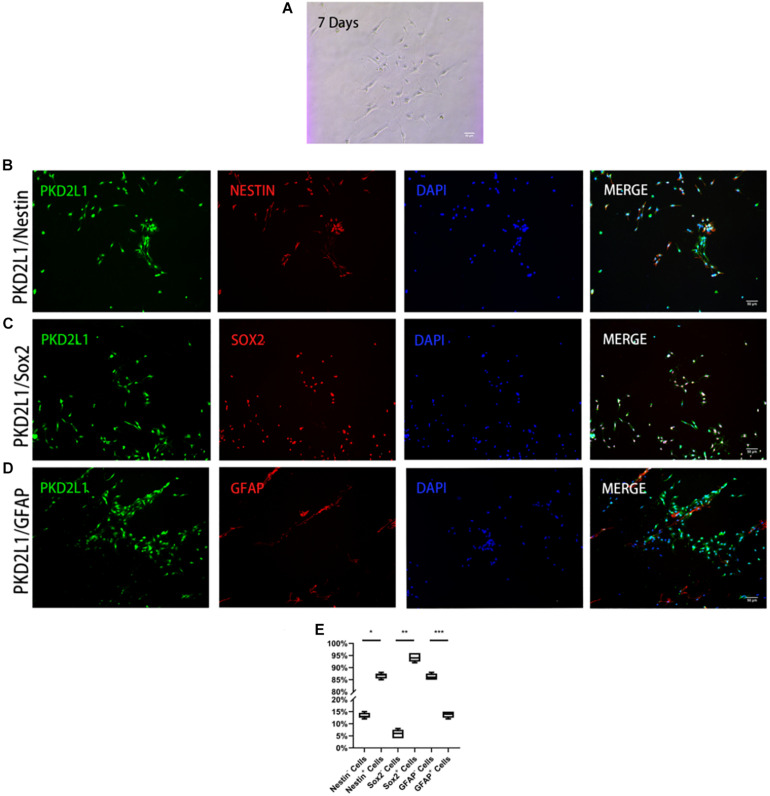
CSF-cNs expressed neural stem cell markers. **(A)** FACS-isolated CSF-cNs were cultured for 7 days. **(B–D)** Immunofluorescence analysis showing that FACS-isolated CSF-cNs expressed neural stem cell markers for nestin **(B)**, Sox2 **(C)**, and GFAP **(D)**. **(E)** The ratios of nestin^+^/nestin^–^ cells, Sox2^+^/Sox2^–^ cells, and GFAP^+^ cells/GFAP^–^ cells, respectively. *P < 0.05 compared to nestin^–^ cells group. **P < 0.05 compared to Sox2-cells group. ***P < 0.05 compared to GFAP^–^ cells group.

Axonal connections among various conglomerates of neurons was observed when CSF-cNs had been cultured for 7 days in an adherent system (Figure 2A). To determine whether these adherent cells exhibited specific stem cell properties, we used immunofluorescence to detect whether these cells expressed markers of NSCs. Analysis showed that approximately 86.60 ± 1.02% of PKD2L1^+^ CSF-cNs expressed Nestin (Figure 2B), 94.30 ± 1.60% expressed SOX2 (Figure 2C), and 13.80 ± 1.17% expressed GFAP (Figures 2D,E).

### CSF-cNs Were Able to Form Neurospheres

**FIGURE 3:**
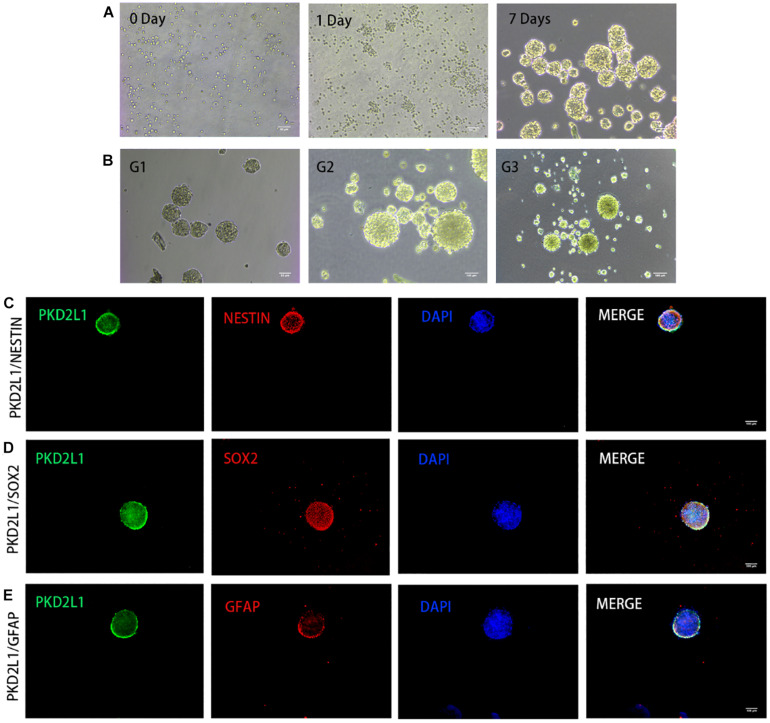
CSF-cNs formed neurospheres and expressed neural stem cell markers. **(A,B)** The growth of neurospheres in different stages. **(A)** 0–7 days in the first passage. **(B)** Passage 1–3. **(C–E)** Immunofluorescence analysis showing that neurospheres expressed neural stem cell markers for nestin **(C)**, Sox2 **(D)**, and GFAP **(E)**.

After 3 days of adherent culture, CSF-cNs were digested by papain and re-inoculated in a Petri dish without a poly-D-lysine coating. At the time of inoculation, a large number of single cells were suspended in the Petri dish and dispersed evenly. One day after inoculation, most of the cells showed mass aggregation; some cells had proliferated while some had fragmented. Seven days after inoculation, most of the suspended cells had proliferated to form neurospheres with good refraction and a strong three-dimensional structure; a small number of cells underwent gradual necrosis as the culture media was changed (Figure 3A). When the cultured neurospheres were transferred for a second passage, their proliferative ability was stronger than that of the original generation. Most cells proliferated, and the time required to form spheres was reduced. After 3–4 days of culture, the diameter of the neurospheres reached approximately 100 mm, although a few cell fragments were still evident. When the neurospheres reached the third passage, the neurospheres had good refraction, a regular shape, and a strong three-dimensional structure. The diameter of these neurospheres ranged from 120 to 150 mm (Figure 3B).

### Neurospheres Formed by CSF-cNs Expressed Neural Stem Cell Markers

Immunofluorescence analysis of the neurospheres from the third passage expressed a panel of typical markers for NSCs, including Nestin (Figure 3C), Sox2 (Figure 3D), and GFAP (Figure 3E). Notably, the neurospheres also expressed PKD2L1, demonstrating the neurospheres formed by CSF-cNs.

### Neurospheres Formed by CSF-cNs Were Able to Proliferate

**FIGURE 4:**
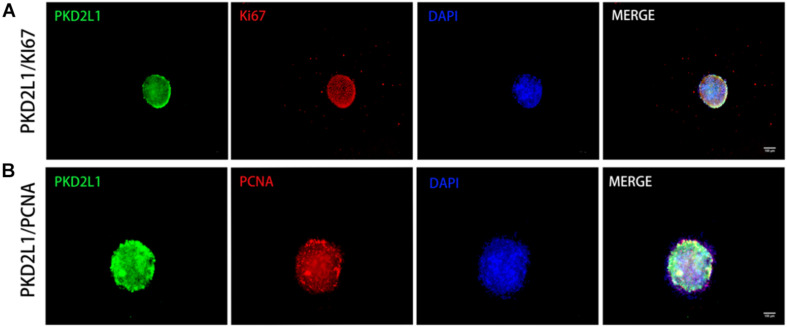
Neurospheres formed by CSF-cNs exhibited the ability to proliferate. **(A,B)** Immunofluorescence analysis showing that neurospheres were able to proliferate and expressed both Ki67 **(A)** and PCNA **(B)**.

Neurospheres formed by CSF-cNs were serially propagated in conventional NSC expansion medium containing fibroblast growth factor-basic (bFGF) and epidermal growth factor (EGF). These cells were highly proliferative. Furthermore, immunostaining detected the expression of the proliferative biomarkers Ki67 (Figure 4A) and PCNA (Figure 4B).

### Neurospheres Formed by CSF-cNs Were Tripotent

**FIGURE 5:**
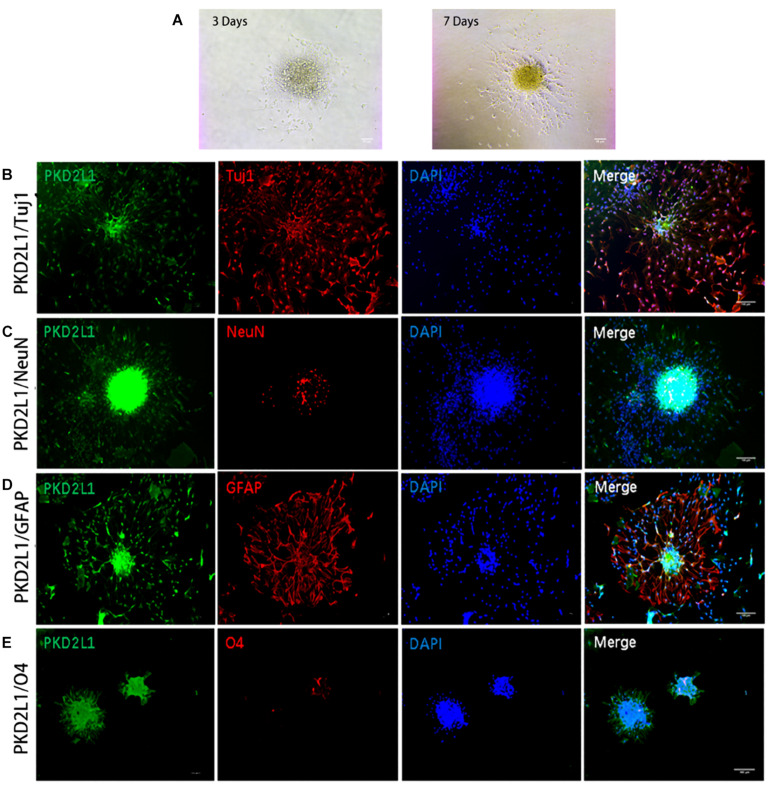
Neurospheres formed by CSF-cNs were tri-potent. **(A)** Differentiation of neurospheres after adherent culture on days 3 and 7. **(B–E)** Immunofluorescence analysis showing that neurospheres differentiated into neurons that were positive for Tuj1 **(B)** and NeuN **(C)**, astrocytes that were positive for GFAP **(D)**, and oligodendrocytes that were positive for O4 **(E)**, respectively. Scale bar represents 50 μm in **(A)** and 100 μm in **(B–E)**.

To characterize the ability of the neurospheres formed by CSF-cNs to differentiate, we re-inoculated neurospheres into a Petri dish coated in poly-D-lysine in a culture media containing bFGF and EGF to encourage differentiation (Figure 5A). After 7 days’ differentiation culture, the cells from the PKD2L1 + CSF-cNs-forming neurospheres expressed Tuj1 and NeuN by immunofluorescence staining, indicating PKD2L1^+^ CSF-cNs has the ability of differentiation into neurons (Figures 5B,C). Next, the expression of GFAP was detected around the neurospheres after 7 days’ differentiation culture by immunofluorescence staining, suggesting that PKD2L1^+^ CSF-cNs is able to differentiate into astrocytes (Figure 5D). In addition, the cells around the neurospheres expressed low of O4 after 7 days’ differentiation culture, implying that PKD2L1^+^ CSF-cNs can differentiate into Oligodentrocytes (Figure 5E). In conclusion, these results demonstrated that the neurospheres formed by PKD2L1^+^ CSF-cNs obtained the potential of differentiation into neoron, astrocyte, and Oligodentrocytes.

## Discussion

Our present study yielded three important findings: (1) CSF-cNs obtained from the tissue surrounding cervical spinal cord central canal of fetal mice expressed markers for NSCs; (2) the CSF-cNs formed neurospheres *in vitro* that also expressed markers for NSCs; and (3) the neurospheres proliferated and differentiated into neurons, astrocytes, and oligodendrocytes. Collectively, these findings indicate that CSF-cNs may represent a form of NSC.

Neural stem cells were first discovered by Reynolds and Weiss (1992). These authors isolated NSCs from the forebrain striatum of adult mammals and successfully used these cells to form neurospheres; these neurospheres were also able to differentiate *in vitro*. The discovery of NSCs overturned our previous belief that the central nervous was unable to regenerate and provides significant new hope for developing protocols to regenerate nerves (Liu et al., 2015). Although significant progress has been made in the field of NSC biology, many questions remain unanswered. The origin of endogenous NSCs has not yet been determined, and the mechanisms involved with the activation of nerve regeneration and repair has yet to be elucidated (Gregoire et al., 2015; Chaker et al., 2016; Li et al., 2016). Therefore, it is vital that we investigate the origin and activation mechanisms associated with endogenous NSCs in adult mammals so that we can fully understand their potential for neurogenesis and regeneration in physiological or pathological states.

Endogenous NSCs grow in a specific NSC niche in the central nervous system that provides a suitable microenvironment for the growth and development of endogenous NSCs. CSF-cNs are a special type of cell within a nest of stem cells. These cells maintain a persistently low state of differentiation and remain persistently immature during development and maturation (Stoeckel et al., 2003; Marichal et al., 2009; Sabourin et al., 2009; Petracca et al., 2016). CSF-cNs also have the same fluid-sensing function as endogenous NSCs (Petrik et al., 2018). We therefore hypothesized that CSF-cNs may be a specific type of NSC. By studying the relationship between fluid-contacting neurons and endogenous NSCs we may be able to gain a better understanding of the composition of endogenous NSCs and their potential for nerve regeneration. Such work may also provide a foundation for research aiming to acquire a more in-depth understanding of neural development and the molecular regulatory mechanisms involved. This may help us to develop new strategies for nerve regeneration and repair.

The cell composition within the NSC niche where CSF-cNs are located is complex. By studying CSF-cNs *in vitro*, we may be able to eliminate interference by the complex *in vivo* environment and gain a better understanding of the functional role of this type of cell in the central nervous system. First, we modified existing culture schemes for purified nerve cells (Brewer and Torricelli, 2007; Mothe and Tator, 2015; Belenguer et al., 2016). Next, we acquired primary nerve tissue from cervical spinal cord of C57BL/6 neonatal mice. This tissue was digested by papain within 24 h. CSF-cNs were subsequently separated and purified by FACS using PKD2L1 a specific marker for CSF-cNs. After FACS, we found that 5.06% of the total cell population were positive for PKD2LI and therefore identified as CSF-cNs. After optimizing the experimental scheme, the survival rate of cells in a single cell suspension was significantly improved. In order to be sure that the isolation procedure is optimized to have purified PKD2L1 + CSF-cNs and no contamination, the adherent cells from culture were detected by immunofluorescence. The result showed that 99.34 ± 0.10% of cells were positive for PKD2L1. We believe that our optimized culture scheme is important for future research as it yields higher cell survival rates, and a purification rate, for CSF-cNs compared with previous studies. This is important as it provides us with a solid foundation for studying CSF-cNs *in vitro.*

NSCs have two basic characteristics: self-renewal and multi-directional differentiation potential. NSCs can maintain self-renewal *via* symmetrical division and can also produce various types of cells that supplement nerve tissue, including neurons, astrocytes, and oligodendrocytes, under appropriate conditions. NSCs that are isolated from primary neural tissue can be cultured in an adherent two-dimensional monolayer culture, or by suspension culture in order to produce clone-derived cell colonies that are referred to as neurospheres (Babu et al., 2007). In order to verify that CSF-cNs exhibit these basic characteristics of NSCs, we separated, purified, and cultured CSF-cNs *in vitro* by adherent culture and by suspension culture so that we could verify their potential as NSCs.

Previous studies have suggested that the markers of NSCs may change during the process of cell lineage transition. During the early stages, NSCs continue to express characteristic stem cell markers. However, during the middle and later stages (7 days later), cells begin to differentiate thus causing NSC markers to transition into other types of marker (Belenguer et al., 2016). We based our protocols on previous literature and cultured purified fluid-contacting neurons in a state of attachment *in vitro*. With increasing culture time, CSF-cNs gradually grew protuberances; most of the cells adopted a spindle-type appearance while a small number of cells were flat in appearance. Immunofluorescence staining showed that at the 3rd day, the primary CSF-cNs expressed high levels of Nestin (86.6%) and SOX2 (94.3%) and the low levels of GFAP (13.8%). These results suggested that CSF-cNs maintain a relatively primitive cell phenotype and show a strong potential as NSCs during *in vitro* culture. Petracca and Colleagues showed that CSF-cNs were negative for GFAP in animal tissues, a marker both for astrocyte and neural stem cell (Petracca et al., 2016; Tonelli Gombalova et al., 2020). But in our research, GFAP was expressed in primary culture of CSF-cNs and neurospheres formed by CSF-cNs in cell culture. Further study should be done to explore the causes of the difference of GFAP expression in tissue and cell culture.

The neurosphere analysis method established by Reynolds and Weiss is recognized as a simple and reliable method for the *in vitro* analysis of neural stem cells, and it is also an effective method with which to verify the self-renewal ability of NSCs (Reynolds and Weiss, 1992). In order to fully evaluate the NSC potential of CSF-cNs, the primary CSF-cNs cells under *in vitro* culture were digested on day 3 with papain and then suspended for further culture (Peng et al., 2019). On the first day of suspension culture, most of the cells showed mass aggregation, although some cells had proliferated and a few cellular fragments were also evident. After 7 days of culture, most of the suspended cells had proliferated to form neurospheres with good refraction and strong stereoscopy.

Neurospheres are a three-dimensional and free-floating population of cells formed in the presence of mitogen and consist of hundreds of cells. A small number of these cells are stem cells; the rest are progenitor cells or mature cells (Morshead et al., 2002; Reynolds and Rietze, 2005). Since each polymer comes from a single cell, only neurospheres that can be passaged and produce cells that can differentiate into neurons, astrocytes, and oligodendrocytes, are considered as NSCs (Richards et al., 1992; Coles-Takabe et al., 2008). Therefore, in this study, the primary neurospheres formed in suspension culture were digested by papain and then sub-cultured. The ability of the newly formed neurospheres to differentiate in the second passage was stronger than that of those in the primary passage; most of the cells underwent proliferation and a shorter time was required for neurospheres to form. After 7 days of culture, most of the cells showed obvious proliferation; most of the neurospheres formed were approximately 100 μm in diameter, although a few cell fragments were still evident. When the neurospheres were passed to the third generation, the neurospheres showed good refraction, a regular shape, and a strong three-dimensional structure; diameters ranged from 120 to 150 μm and the neurospheres exhibited a strong ability to self-renew. Next, we selected some neurospheres from the third passage that showed good rates of growth and used immunofluorescence to demonstrate that the cells in these spheres expressed high levels of PKD2L1. Furthermore, we demonstrated that the neurospheres also expressed established markers for NSCs (Nestin, Sox2, and GFAP) and known markers for cell proliferation (Ki67 and PCNA), thus exhibiting the characteristics of early NSCs with strong cell proliferation activity.

Neural stem cells not only exhibit a strong ability for self-renewal, they also need to have the ability to differentiate into neurons, astrocytes, and oligodendrocytes. Therefore, we also selected well-growing neurospheres from the third generation and inoculated them into a Petri dish coated with poly-D-lysine to induce adherent differentiation. After 7 days of culture, the cells surrounding the neurospheres spread out in all directions; the morphology of glial cells and neurons was also more apparent. Immunofluorescence results on day 7 showed that the neuronal immature marker Tuj1, and astrocyte marker GFAP, were strongly expressed in the adherent differentiated neurospheres; low levels of the mature neuronal marker NeuN, and the oligodendrocyte marker O4, were also expressed. The results arising from our immunofluorescence analysis were consistent with the characteristics of NSC differentiation.

In this research, we used established methods to prove that when cultured *in vitro*, CSF-cNs are able to undergo self-renewal and show multi-differentiation potential, thus exhibiting the basic characteristics of NSCs. However, there were some limitations to our research that also need to be considered. Firstly, *in vitro* cell culture was used to evaluate the NSC characteristics of CSF-cNs, consequently these cells had been separated from their original microenvironment. It is therefore possible that the *in vitro* environment did not fully replicate the endogenous environment for these cells *in vivo*. Secondly, following cell differentiation, we did not perform any electrophysiological examinations of the cells; therefore, we were not able to examine the functionality of these cells after differentiation (Zhang et al., 2016). These limitations may have a certain impact on the reliability of our results and should be addressed in future research.

## Conclusion

In conclusion, experiments involving neurosphere formation and induced differentiation *in vitro* clearly demonstrated that CSF-cNs have the potential for self-renewal and multi-directional differentiation when cultured in suspension. It is evident that CSF-cNs are similar to NSCs in the central nervous system. Previous studies have also suggested that this population of cells maintain a persistently low state of differentiation during adulthood. We also demonstrated that when cultured *in vitro* CSF-cNs exhibit the two major characteristics of neural stem cells. Collectively, PKD2L1^+^ CSF-cNs have the property of neural stem cell *in vitro*. Our next research will be done to confirm the PKD2L1^+^ CSF-cNs as neural stem cell *in vivo* and to explore its role in the spinal cord function.

## Data Availability Statement

The original contributions presented in the study are included in the article/supplementary material, further inquiries can be directed to the corresponding author/s.

## Ethics Statement

The animal study was reviewed and approved by the Animal Care Welfare Committee of Guizhou Medical University.

## Author Contributions

QL and XD designed the experiments. SW, HY, LCh, WT, QT, and YP performed the experiments. SW, YH, and HZ performed the informatics analysis. YH and SW wrote the manuscript. All authors contributed to the article and approved the submitted version.

## Conflict of Interest

The authors declare that the research was conducted in the absence of any commercial or financial relationships that could be construed as a potential conflict of interest.
